# Three-way multiplexing in prostate cancer patients — combining a bimodal sentinel node tracer with multicolor fluorescence imaging

**DOI:** 10.1007/s00259-022-06034-x

**Published:** 2022-11-19

**Authors:** Anne-Claire Berrens, Matthias N. van Oosterom, Leon J. Slof, Fijs W. B. van Leeuwen, Henk G. van der Poel, Tessa Buckle

**Affiliations:** 1 Department of Radiology, Interventional Molecular Imaging Laboratory, Leiden University Medical Centre, Albinusdreef 2, Leiden, 2300 RC The Netherlands; 2 Department of Urology, Netherlands Cancer Institute – Antoni van Leeuwenhoek Hospital (NKI-AVL), Amsterdam, The Netherlands; 3 Department of Design and Prototyping, Leiden University Medical Center, Leiden, The Netherlands; 4 Department of Urology, Amsterdam University Medical Center, VUmc, Amsterdam, The Netherlands

We present images from a 71-year-old prostate cancer patient who underwent a robot-assisted radical prostatectomy and extended pelvic lymph node dissection (ePLND) complemented with experimental sentinel node (SN) resection [1]. Additional intraoperative lymphangiography was performed to highlight lymphatic structures that should ideally be spared. This concept is part of a prospective trial wherein we aim to use the presented approach to reduce the 20% complication rate associated with ePLND procedures [2].

For SN identification, the bimodal/hybrid tracer ICG-^99m^Tc-nanoscan (218 MBq in 2 ml) was used, which has replaced the well-known ICG-^99m^Tc-nanocolloid [3]. Preoperative (lymphoscintigraphy and SPECT/CT) and intraoperative SN imaging (gamma and fluorescence imaging) were performed after intraprostatic tracer injection. Previous work indicates that the hybrid tracer and the visible-dye fluorescein both stain lymphatic structures, but with different kinetics [4]. It is also known that multi-color fluorescence imaging can be used to differentiate between lymphatics of different organs [5, 6]. With this first-in-human implementation (see Scheme), we underscore that lymphatic ducts draining the left upper leg can be made visible using fluorescein (80 mg in 4 ml; yellow, dotted triangle) and that this drainage can be distinguished from the SNs visualized with ICG-^99m^Tc-nanoscan (blue, dotted circle). With that, the former can act as “red-flag” to highlight tissues that should be spared, while the latter provides a ”green-flag” for tissues that should be resected. As such, the presented three-way multiplexing approach may help preserve the oncological benefit of ePLND + SN [3], while providing a handle to reduce complication rates.
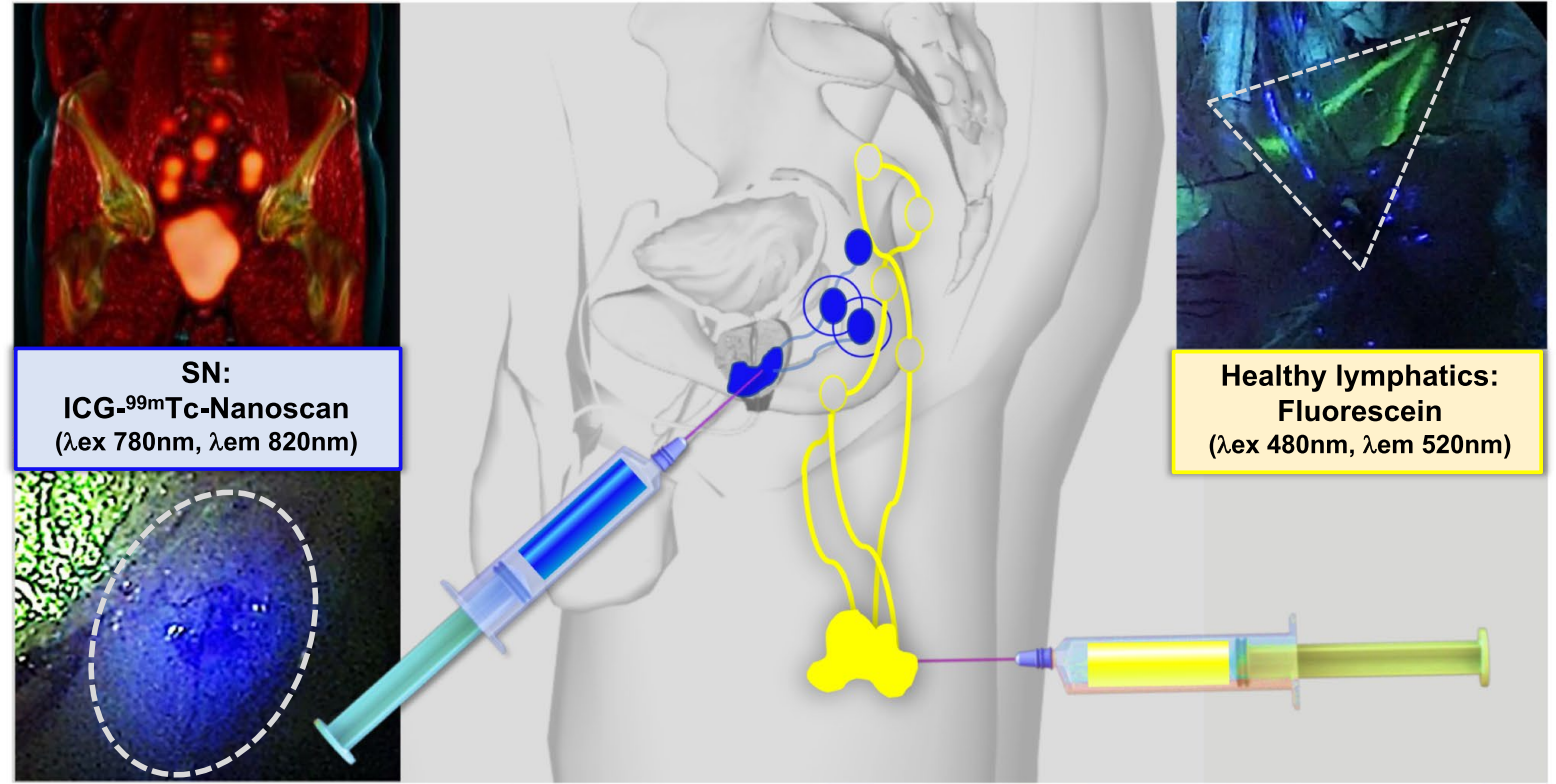


## References

[1] Wit EMK, Acar C, Grivas N, Yuan C, Horenblas S, Liedberg F (2017). Sentinel node procedure in prostate cancer: a systematic review to assess diagnostic accuracy. Eur Urol.

[2] Briganti A, Chun FK, Salonia A, Suardi N, Gallina A, Da Pozzo LF (2006). Complications and other surgical outcomes associated with extended pelvic lymphadenectomy in men with localized prostate cancer. Eur Urol.

[3] Mazzone E, Dell'Oglio P, Grivas N, Wit E, Donswijk M, Briganti A (2021). Diagnostic value, oncologic outcomes, and safety profile of image-guided surgery technologies during robot-assisted lymph node dissection with sentinel node biopsy for prostate cancer. J Nucl Med.

[4] van den Berg NS, Buckle T, KleinJan GH, van der Poel HG, van Leeuwen FW (2016). Multispectral fluorescence imaging during robot-assisted laparoscopic sentinel node biopsy: a first step towards a fluorescence-based anatomic roadmap. Eur Urol.

[5] Kobayashi H, Longmire MR, Ogawa M, Choyke PL, Kawamoto S (2010). Multiplexed imaging in cancer diagnosis: applications and future advances. Lancet Oncol.

[6] Meershoek P, KleinJan GH, van Willigen DM, Bauwens KP, Spa SJ, van Beurden F (2021). Multi-wavelength fluorescence imaging with a da Vinci Firefly—a technical look behind the scenes. J Robot Surg.

